# Research on Response Parameters and Classification Identification Method of Concrete Vibration Process

**DOI:** 10.3390/ma16082958

**Published:** 2023-04-07

**Authors:** Yuanshan Ma, Zhenghong Tian, Xiaobin Xu, Hengrui Liu, Jiajie Li, Haoyue Fan

**Affiliations:** 1 College of Water Conservancy and Hydropower Engineering, Hohai University, Nanjing 210098, China; 2 State Key Laboratory of Hydrology Water Resources and Hydraulic Engineering, Hohai University, Nanjing 210098, China; 3 College of Mechanical & Electrical Engineering, Hohai University, Changzhou 213022, China

**Keywords:** vibrating process, vibration acceleration signal, deep learning, quality evaluation method

## Abstract

The vibration process applied to fresh concrete is an important link in the construction process, but the lack of effective monitoring and evaluation methods results in the quality of the vibration process being difficult to control and, therefore, the structural quality of the resulting concrete structures difficult to guarantee. In this paper, according to the sensitivity of internal vibrators to vibration acceleration changes under different vibration media, the vibration signals of vibrators in air, concrete mixtures, and reinforced concrete mixtures were collected experimentally. Based on a deep learning algorithm for load recognition of rotating machinery, a multi-scale convolution neural network combined with a self-attention feature fusion mechanism (SE-MCNN) was proposed for medium attribute recognition of concrete vibrators. The model can accurately classify and identify vibrator vibration signals under different working conditions with a recognition accuracy of up to 97%. According to the classification results of the model, the continuous working times of vibrators in different media can be further statistically divided, which provides a new method for accurate quantitative evaluation of the quality of the concrete vibration process.

## 1. Introduction

Reinforced concrete is the most used building material in the world today. The selection of appropriate raw materials and the use of appropriate technology can lead to strong building structures [1,2,3]. Among various construction technologies, the vibration process is an indispensable and important procedure in the construction process. In this, vibration is applied to the concrete mixture to remove trapped air and promote the relative movement and redistribution of each component, thus improving the compactness of concrete [4,5].

However, insufficient vibration time causes problems such as honeycomb structure, voids, and exposed reinforcement in the concrete mixture, while too long a vibration time causes segregation and the uneven distribution of aggregates [6,7]. This may cause significant problems, such as a decline in mechanical strength, increased shrinkage and cracking, and the reduction of chemical erosion resistance, all of which are detrimental to the performance of reinforced concrete structures [8,9,10]. Due to the hidden nature of the concrete interior, the above problems are difficult to detect during vibration construction. It is only after the concrete has hardened and the external formwork has been removed that the various quality problems caused by inefficient vibration processing are exposed, and the repair of these problems in quality is difficult and time-consuming. Although construction specifications place specific requirements on the vibration process [11], there is still a lack of accurate quality evaluation methods, and the quality of vibration construction depends largely on the experience and subjective judgment of workers. Therefore, it is urgent to establish a method for closely monitoring the vibration process that can quantify the quality of vibration processing using relevant data.

Taking internal vibrator vibration construction as an example, the single vibration process of concrete mixtures is mainly divided into two stages: vibrator insertion and pull-out. The key to vibration process monitoring is to determine the stage in which the vibrator is working, and the duration of vibrator working in each stage is recorded to obtain the actual energy acting on the concrete mixture and quantify the quality of the concrete vibration processing. Burlingame [12] believed that the internal vibrator could provide local heating of the concrete with which it was in contact, and the working state and position of the vibrator could be determined by infrared thermal imaging. However, the accuracy of this method was low, and it was difficult for thermal imaging to penetrate the concrete when the structure size was large. Li et al. [13] proposed a positioning device for concrete vibrators based on laser positioning, which can send time signals to a laser detector by triggering changes in laser emissions, but this device can only record the vertical movement of a vibrator, and cannot judge whether the vibrator vibrates inside the concrete. Wang et al. [14] used an infrared sensor to identify the insertion depth of a vibration trolley. Zhong et al. [15] installed an ultrasonic sensor on the vibration trolley to measure the distance between the vibrator and the concrete and calculate the duration of vibration. However, the ranging sensor easily became covered by cement slurry resulting in a loss in accuracy. Liu [16] proposed a method to track and identify the position and state of the internal vibrator in real-time through stereo cameras, but the stereo camera system required complex and time-consuming manual calibration in the field, which was hindered by the lighting conditions in the field. Li et al. [17] developed a device and method for determining the vibrator operating state (insertion or pull out) according to the variation in input current of the drive motor of the internal vibrator when inserted and pulled out of the concrete. Tian et al. [18] proposed an intelligent measurement method for vibration duration, which used the difference between the working current and the vibrating audio frequency of the vibrator to determine whether the vibrator was in a vibrating state through an intelligent module. This study successfully confirmed the method of determining the vibrator operating state and recording the vibration time, which can be used to parameterize the vibration quality. However, the current signal of the equipment was easily affected by the stability of the supply voltage and electromagnetic signal interference of other mechanical equipment, and the degree of refinement was difficult to ensure.

The above studies were limited to the monitoring of the plain concrete vibration process. In actual engineering, concrete structures usually included steel mesh to improve mechanical properties [19], and the actual working efficiency of vibrators varies in different vibration media. Forssblad [20] and Zhang [21,22] found that steel bars significantly blocked the propagation of excitation force in the vibration process of mixtures, and it was necessary to improve the vibration efficiency and extend the vibration time when vibrating reinforced concrete. However, at present, the vibration process has not put forward special requirements for reinforced concrete. Therefore, the vibrator insertion stage of a single vibration process needs to be finely divided to determine whether the concrete has reinforcement or not, before quantitatively evaluating the quality of the vibration processing of the concrete.

In order to solve the above problems, this paper studied and determined the sensitivity of acceleration signals to high-frequency vibration, and a vibration medium identification method based on vibration acceleration signals of concrete vibrators was proposed. A multi-scale convolutional neural network (MCNN) was used to classify the vibrator acceleration signals, which can identify the stages of the vibration process and accurately classify the duration of the vibration process. This paper has provided a new basis for the quantitative evaluation of the quality of vibration processing and has helped to monitor potential construction quality problems.

## 2. Materials and Methods

### 2.1. Principle of Vibration Medium Identification

**Figure 1:**
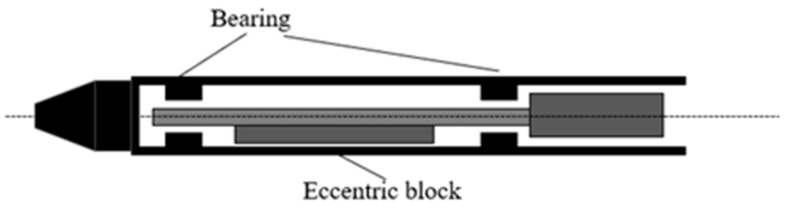
Internal vibration structure diagram of an eccentric vibrator.

**Figure 2:**
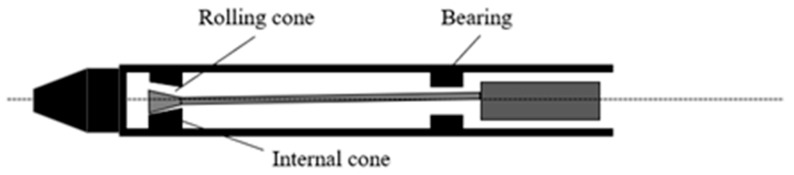
Internal vibration structure diagram of a pendulum vibrator.

An internal vibrator is widely used and suitable for various types of concrete projects. According to structural composition and vibration mode, vibrators can be divided into pendulum-type and eccentric-type vibrators. The internal initiation structures of the two types of vibrators are shown in Figure 1 and Figure 2. When the pendulum vibrator works, it uses the rotating axis of the rolling body on the conical surface inside the vibrating rod to roll and form planetary motion to drive the rod to produce vibration. The eccentric vibrator uses the centrifugal force of the high-speed rotation of the internal mass eccentric shaft as the exciting force, which is transferred to the vibrator rod shell by the bearing. By studying the motion equations of the vibration process of the two types of vibrators, it is found that both are simple harmonic vibrations with the same frequency and amplitude along the radial direction of the vibrator rod head, which can be represented by a sinusoidal waveform (Equation (1)).

(1) X=Asinwt=Asin2πft

where *X* is rod head displacement, mm; *A* is amplitude, mm; *f* is the frequency, Hz; and *t* is time, s.

Vibration is a dynamic quantity, and simple harmonic vibration is the simplest form of vibration. In addition to vibration displacement, vibration velocity and acceleration are often used in vibration analysis [23]. The vibration acceleration (Equation (2)) can be obtained by calculating the second derivative of the displacement signal with respect to time. The amplitude of vibration acceleration is the product of vibration displacement and frequency squared, and the amplitude reflects the influence of the vibration frequency and displacement amplitude at the same time, which is more comprehensive than the simple vibration displacement amplitude. The more obvious the “amplification” effect of the high-frequency component of the acceleration spectrum, the more effective the vibration acceleration monitoring in the high-frequency vibration analysis.

(2) $$a=X^{\prime \prime }=w^{2}A\sin(wt+\pi )$$

In the process of concrete vibration, the vibrator generated high-frequency vibration when working, producing energy transfer inside the concrete. According to the American Concrete Institute (ACI) research report ACI 309 [24], the formula for concrete vibration energy is given by (Equation (3)):

(3) $$E=CmA^{2}f^{2}t$$

where *E* is vibration energy, J; *C* is a constant, depending on the stiffness and damping of concrete; *m* is the mass of concrete under vibration, kg; *A* is the maximum amplitude of concrete, *m*; *f* is vibration frequency, Hz; and *t* is the vibrating time, s.

Equations (1) and (2) can be obtained as follows:

(4) $$a=4\pi^{2}f^{2}A$$

By combining Equations (3) and (4), the vibration energy per unit time can be written as:

(5) $$E_{0}=\frac{Ca_{b}{}^{2}}{16\pi^{4}f}$$

where *a_b_* is the acceleration of the distance between the shaken concrete and the vibrator b m, m/s^2^.

According to the literature [25,26], the relationship between the vibrator and the internal acceleration of concrete is as follows:

(6) $$a_{b}=\frac{a_{0}\xi_{0}e^{-\xi_{m}b}}{\sqrt{b}}$$

where *ζ*_0_ is boundary damping, *ζ*_m_ is material damping, and *a*_0_ is vibrator acceleration.

According to Equations (5) and (6) and simplified constant terms, Equation (7) can be obtained:

(7) $$E_{0}=C_{0}a_{0}{}^{2}$$

This represents the total damping of the vibration system.

According to Equation (7), when the vibrator is unloaded, the damping is small and the vibrator acceleration reaches its peak. When the vibrator is inserted into the concrete, the damping increases, causing the vibrator acceleration to decrease. At the same time, the existence of steel bars in concrete presents obvious obstacles to the propagation of vibration energy, which leads to a further increase in the vibration system damping and the corresponding reduction in vibrator acceleration. According to the research of Alexander [27], He [28], and Yuan [29], acceleration generated by the vibrator is the main reason for the internal state transformation of the concrete mixture [30], so acceleration is the main factor in vibration compaction. Meanwhile, the cumulative energy represented by the vibrator acceleration is a decisive factor in the solid-liquid state transition of the mixture.

When the vibrator is working, it interacts with the mixture, and the properties of the mixture medium are also fed back to the vibrator and characterized by the change in vibration parameters. Therefore, for the analysis and study of the concrete vibration process, the vibrator acceleration is selected as the main parameter to reflect the vibrator’s working state more sensitively. Based on this, the method of accurately identifying the properties of the vibration medium is proposed.

### 2.2. Test Device

**Figure 3:**
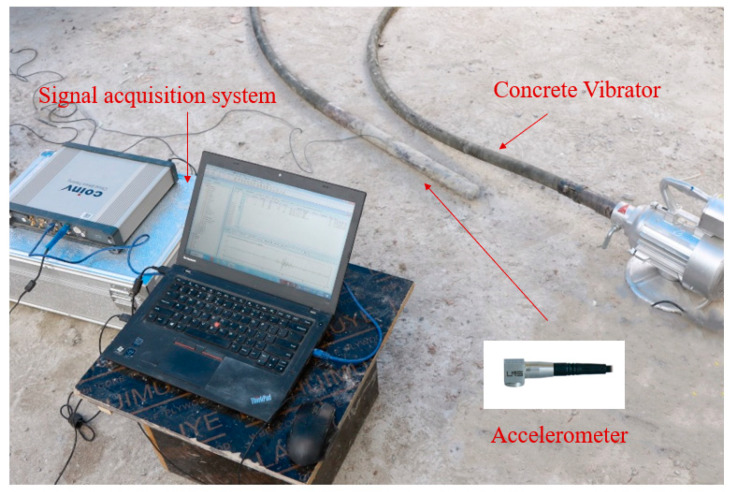
Signal Acquisition System and Accelerometer.

The mechanism between the vibrator and concrete in the process of concrete vibration was described in detail in Section 2.1, which also provided a theoretical basis for the vibration acceleration signal to identify the properties of the medium under vibration. In order to obtain the vibration acceleration signal during vibration, a device for the real-time acquisition of the acceleration of the vibrator rod head was designed in this paper. The equipment consisted mainly of a signal acquisition instrument and an IEPE uniaxial watertight acceleration sensor. The device connections are shown in Figure 3. The sampling rate of the acquisition instrument is adjustable from 10–200 kHz, and the sensor is rigidly connected to the vibrator and has a high installation resonance frequency. The installation and connection of the test device is simple and suitable for various types of vibrators.

The above device is used to measure the vibration acceleration data of 50 mm diameter vibrators of different brands (only differentiated by place of origin). The vibration signal is simply analyzed, and the main frequency of vibration is obtained through spectrum analysis. According to Equations (1) and (2), in simple harmonic vibration, the amplitude (maximum displacement X max) and acceleration can be interconverted and have the same frequency, which can reflect the amplitude of vibration. However, according to the measured parameters listed in Table 1 and the parameters listed on the vibrator nameplate, it is found that the test acceleration under the same listed amplitude and the main frequency of vibration under the same motor speed are very different, which is closely related to the internal structure, structural materials, assembly mode and driving motor of the vibrator [31,32].

When the vibrator vibrates in reinforced concrete, it is difficult to obtain accurate data for variable load vibration signal analysis by traditional vibration analysis methods such as amplitude analysis, frequency spectrum analysis, phase analysis, and waveform analysis in the face of heterogeneous media. The physical model and vibration signal identification method rely on expert experience, which cannot meet the real-time, fast, and accurate identification requirements of concrete vibration technology.

### 2.3. Vibration Signal Recognition Method of Multi-Scale Convolutional Neural Network Combined with Attention Mechanism (SE-MCNN)

With the development of sensor technology and information technology, the use of machine learning algorithms provides a new solution for mechanical intelligent fault diagnosis because of their powerful data processing ability. According to the depth of the network structure, machine learning can be divided into either shallow or deep learning [33]. Deep learning [34] is more suitable for mechanical vibration state monitoring and recognition because of its powerful depth feature extraction ability. Common deep learning methods mainly include the convolutional neural network (CNN) [35], autoencoder (AE) [36], deep confidence network (DBN) [37], etc. Among them, the convolutional neural network (CNN), as a typical deep neural network, has a strong ability to independently extract features [38].

A concrete vibrator is a rotating machine that generates vibration through the internal rotor rotating at high speed. For the identification of the vibration medium in the vibration process, concrete as a vibration medium is not uniform, and the addition of reinforcement can make the load change, and a single-scale convolution kernel is not conducive to learning vibration signal characteristics under load change. If only a single-scale convolution kernel is used in a single-layer convolution layer, other features of different fineness are easily ignored. A multi-scale convolutional neural network (MCNN) can extract features of different fineness from complex signals, considering the multi-scale characteristics displayed by vibration signals [39]. In order to extract data features more comprehensively, an MCNN model is used to classify and recognize vibration signals. A multi-scale convolution layer sets convolution kernels of different scales for the convolution operation, avoiding the ignoring of features of different fineness of a single-scale convolution check, which can enhance the network’s ability to express features and realize the finely distributed characterization of vibration signal features in the time domain [40,41].

**Figure 4:**
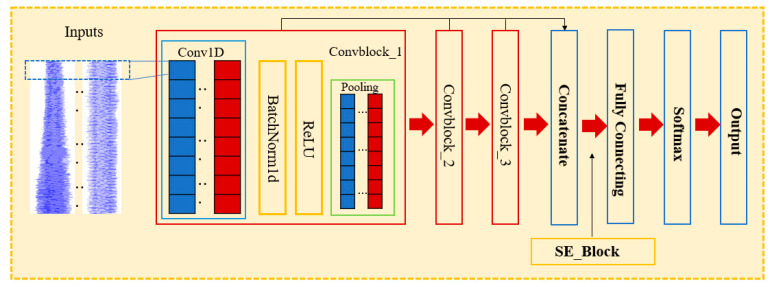
SE-MCNN Model structure diagram.

Therefore, a multi-scale convolutional neural network combined with a self-attention feature fusion mechanism (SE-MCNN) was proposed to identify the vibration medium. The self-attention mechanism was used for the weighted fusion of multi-scale features to obtain the final features that can accurately identify the medium type. The structure of the SE-MCNN model is shown in Figure 4. The original vibration signal of the vibrator was taken as the input of the model, and the characteristic information of the vibration signal was extracted through the MCNN module. Then, the attention mechanism was used to assign weights to the characteristic information to improve the degree of attention of the model to the key information. Finally, the recognition and classification of the vibration medium of the vibrator was completed through the fully connected layer.

**Figure 5:**
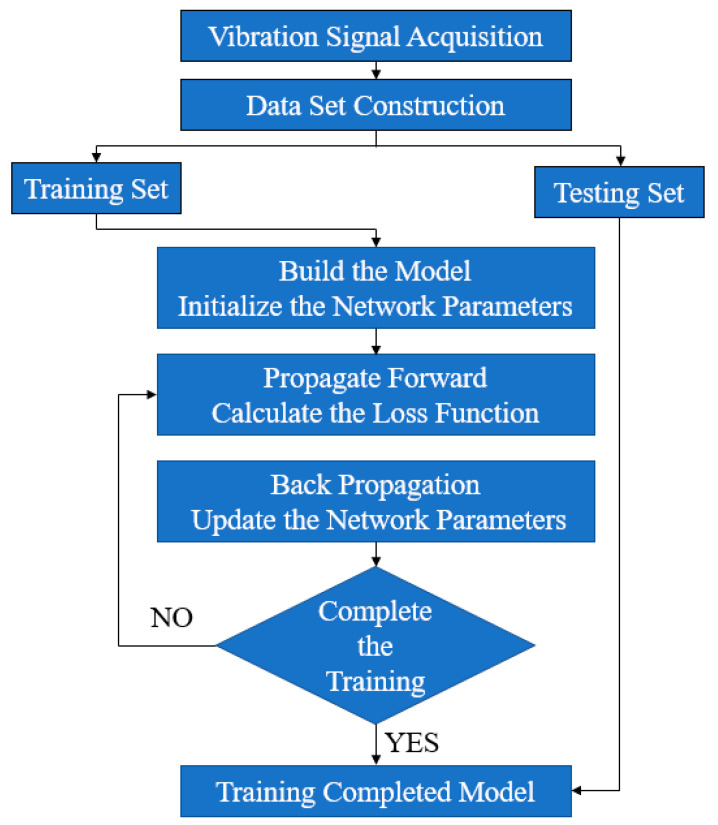
Vibration signal recognition process based on a SE-MCNN model.

The media identification process based on SE-MCNN is shown in Figure 5. The fault diagnosis steps are as follows:

Step 1: Sensors are used to collect vibrator vibration signals under different vibration media, and the signals are truncated into individual samples and labeled.

Step 2: Labeled samples are randomly divided into training sets and test sets. The training set is used to train the SE-MCNN network. The loss function of the model optimizes the model parameters through gradient backpropagation. After several iterations, model training is completed.

Step 3: The test set is input into the trained SE-MCNN model to identify the type of vibration medium of unknown signals, and the recognition accuracy is calculated through known labels to verify the effectiveness of the model.

### 2.4. Test Scheme

**Figure 6:**
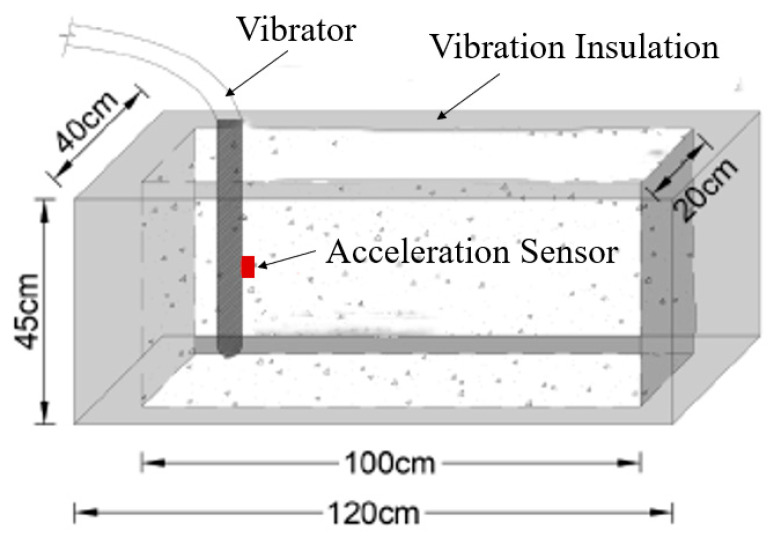
Test mold schematic diagram.

**Figure 7:**
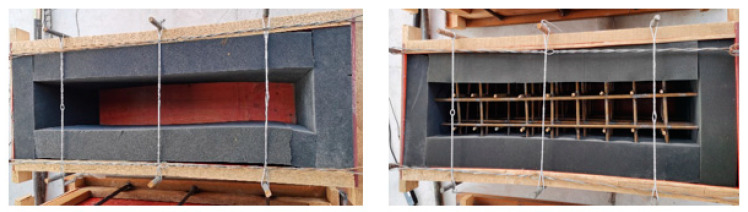
Actual test mold.

**Figure 8:**
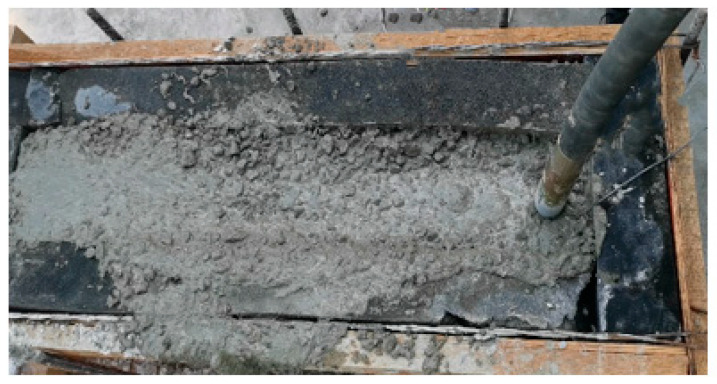
Test process.

In order to verify the feasibility of the above method and the accuracy of model recognition, an experiment was designed. The specific test scheme was as follows: Two wooden molds were made and vibration isolation sponges were pasted inside to eliminate the influence of the template-reflected vibration waves on the test. The structure size is shown in Figure 6. The vibration signal acquisition devices were connected to the five different internal vibrators listed in Table 1 and vibration signals under different working conditions were collected. According to the actual situation of the concrete vibration process at the engineering site, the different vibration media can be divided into air, concrete mixture, and reinforced concrete mixture, as shown in Figure 7. The air medium is the vibrator no-load, corresponding to the vibrator pull-out state in the vibrating process, while the remaining two media correspond to the states in which the vibrator is inserted into the mixture of pure concrete and reinforced concrete, as shown in Figure 8. In order to show the universality of the test, the concrete is configured with the most commonly used mix ratio, which is 0.5 w/c ratio and 150 mm slump, and the specific mix ratio is given in Table 2. In the case of reinforced concrete, the steel mesh in Figure 7 was arranged at 10 cm intervals.

The test was divided into five groups according to the different vibrators. In each group of tests, the concrete in the mold is poured out and re-stirred after the end of the first vibration to ensure that the fluidity of the mixture in the second test was the same as that in the first test. During the test, each vibrator was vibrated in each medium for 30 s to ensure that sufficient vibration signals were collected for MCNN model training and verification.

## 3. Test Results and Verification

The vibration signals collected above were de-noised, then normalized and labeled. Finally, the dataset was divided into training and test sets according to a 7:3 ratio. This was used to train and test the SE-MCNN recognition model, and the recognition accuracy was 96.1%. According to the identification results of the vibrator vibration medium, the vibration process stage of the vibrator can be finely divided, which provided the basis for accurate quantitative evaluation of vibration quality.

### 3.1. SE-MCNN Model Parameters

Vibration signals usually exhibit multi-scale characteristics. In the SE-MCNN model, convolution layers with different kernel sizes are used to extract the multi-scale information of the vibrator signals. However, the increasing number of scales not only brings more information to the model but also increases the complexity and training difficulty of the network, resulting in unnecessary calculation loss. In addition, with the deepening of the fault diagnosis model and the increase in convolutional modules, the extracted features become more separable. However, the deepening of the model will inevitably complicate the modeling and easily lead to overfitting.

In this paper, the scale and depth of the model framework were determined to be 3 after several iterations of training and optimization. Detailed model parameters are shown in Table 3. Mean square error was selected as a loss function to measure the similarity between the predicted Softmax output probability distribution and the target class probability distribution, and the Adam optimizer was used to adjust the learning rate adaptively. The batch size is 500, the learning rate is 0.001, and the number of iterations is 800. The model runs in Python+Keras, and the hardware environment utilizes an Inter i7-8700 CPU and an Nvidia GeForce GTX 1080 Ti graphics card.

### 3.2. Verification Results of SE-MCNN Model

**Figure 9:**
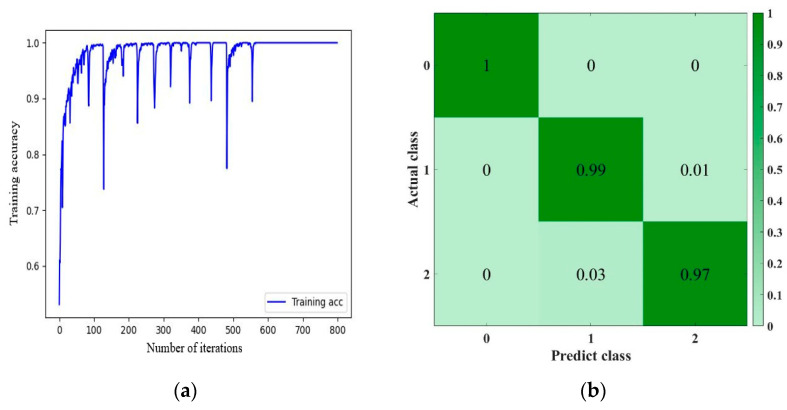
The learning curve and confusion matrix of the SE-MCNN model. (**a**) Training curve. (**b**) Test set confusion matrix.

In order to evaluate the recognition ability of the network after training, Figure 9b shows the confusion matrix used to check the recognition results. Where, “0”, “1” and “2” indicate that the vibrator is in the “pulled-out state”, “inserted into plain concrete state” and “inserted into reinforced concrete state”, respectively. The state identification accuracy is 100%, 99%, and 97%, respectively. Since the damping of the concrete mix after vibration has time-varying characteristics, and the flow state of the mixture may change when the vibrator is inserted into reinforced concrete, the recognition accuracy of the “1” and “2” states is slightly lower, but the overall recognition accuracy is still at a high level. It can be seen from the training process line (Figure 9a) that although there are fluctuations in the early stage of the training process of the data set, the convergence rate is relatively fast. Therefore, the model can easily identify and classify the vibration medium of the vibrator and has good stability.

### 3.3. Fine Division of Vibrating Process Stage and Duration Statistics

Due to the time-sensitivity of the concrete vibration process, many researchers [6,42,43,44,45] have tried various methods to determine the state of vibrator insertion and removal and record the vibration time to ensure the quality of concrete vibration construction. However, because concrete structures are very complex, the working state of the vibrator is likely to change in a very short time in the actual construction process—that is, there is rapid, continuous insertion and pull-out of the vibrator over a short time. It is difficult for existing equipment to accurately and completely record the actual time of each vibration stage. In addition, the vibration time of reinforced concrete is not recorded separately in order to evaluate the construction quality of the vibration process more accurately. In this section, the tests are designed to simulate the site construction conditions, and the actual duration of each stage of vibration is calculated statistically based on the output results of the model to quantitatively evaluate the construction quality of the whole process of vibration.

**Figure 10:**
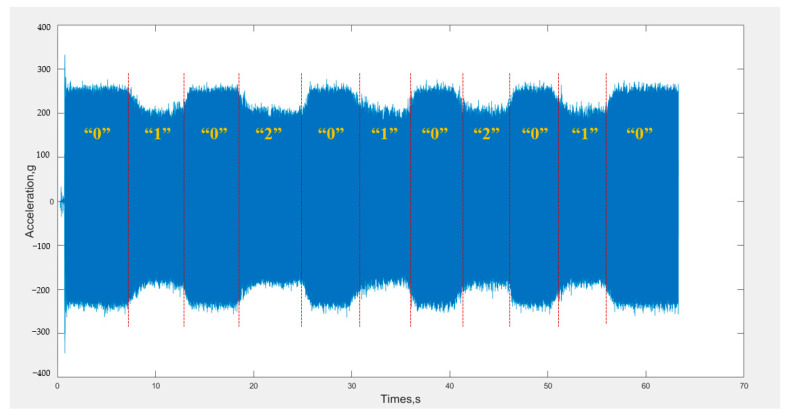
Time domain diagram of the acceleration signal.

Concrete was poured into the two wooden molds described in Section 2.4, and an internal vibrator was randomly selected and connected to an acceleration sensor. The construction site vibration process was simulated, and the plain concrete and reinforced concrete mold were respectively vibrated for 5 s before pulling out, and this process was repeated five times at random insertion points. The cumulative vibration time of plain concrete mold insertion was about 15 s, and the cumulative vibration time of reinforced concrete mold insertion was about 10 s. An example of the collected vibration acceleration signals is shown in Figure 10.

The above vibration signals were input into the trained SE-MCNN model for recognition and classification. The model divided the batch size of vibration signals into 500. As the sampling frequency was 20,000 Hz, the duration of each group of signal points was 0.025 s. The classification results are listed in Table 4. Although there were errors in the simulation of vibration construction timing, the duration of each state after model classification was largely consistent with the actual duration, which indicated that the method had a very high precision for the classification of vibration process duration. According to the formula for concrete vibration energy (Equation (7)), the following formula can be obtained by multiplying the assigned vibrator acceleration with the vibration duration under different media:

(8) $$W=\sum_{i=0,1,2}^{}E_{0}t_{i}=\sum_{i=0,1,2}^{}C_{0}a_{0}t_{i}$$

where *W* is the accumulated energy of vibration and *t_i_* is the duration of vibration in each stage.

Thus, the cumulative vibration energy can be calculated according to the amplitude of the real-time acceleration signal and the vibration duration of each stage after classification. Combined with the energy threshold [26], the quality of concrete vibration construction can be accurately and quantitatively evaluated.

## 4. Conclusions

This paper classified and identified vibrator acceleration signals and finely divided the vibrating process into different stages. The following conclusions can be drawn and prospects identified:

1. Based on the characteristics of the vibration acceleration signal for comprehensive and effective monitoring of high-frequency vibration, this paper studies the ability of acceleration to characterize the medium attribute information of concrete in the process of vibration and determines the direct correlation and sensitivity of vibrator acceleration signals to the identification and analysis of the medium under vibration.
2. Through the introduction of a vibration signal analysis method, a multi-scale convolutional neural network combined with a self-attention feature fusion mechanism (SE-MCNN) was proposed to identify the vibration medium, which can accurately identify and classify the vibrator “pull-out”, “insertion into plain concrete”, “insertion into reinforced concrete” three main vibration process stages. The identification accuracy of this method was up to 97.0% and the stability was good.
3. According to the recognition and classification results of model vibration signals, the complete vibration construction process is finely divided into different stages, and the actual working duration of vibrators at each stage is calculated using statistical signal time-domain information. The vibration duration at different stages is combined with the vibration energy formula to calculate the parameters of the actual vibration energy absorbed by the concrete at each stage. Combined with the threshold parameters of vibration energy, a precise quantitative evaluation method for concrete vibration quality is proposed.
4. The application of the model and method can not only monitor the working state of the vibrator but also provide quantitative statistical parameters of the quality of the vibration processing for practical engineering, plugging a gap in field vibration quality evaluation. If the proposed method is combined with wireless communication, accurate real-time quality monitoring and feedback of the vibration process can be provided for on-site construction quality supervisors to ensure high-quality construction utilizing concrete vibration.

## Figures and Tables

**Table 1:** Vibrating rod parameters.

NO.	Production Place	Motor Form	Nameplate Parameter Motor Speed/Amplitude (rpm/mm)	Main Vibrating Frequency (Hz)	Measured Acceleration (g)
1	Germany	Built-in Motor	12,000/1.2	190	200
2	Japan		12,000/1.0	195	180
3	Guangdong, China		12,000/1.0	185	150
4	Guangdong, China	External Motor	4000/1.5	243	220
5	Shandong, China		2850/1.5	210	200

**Table 2:** Concrete mix.

Mixture Proportions (kg/m^3^)					W/C
W	C	S	G	SP	
230	460	676	1040	2.30	0.5

Notes: W, C, and S indicate water, ordinary Portland cement P.O. 42.5, and river sand, respectively; G represents crushed stone with a minimum and maximum diameter of 5 and 20 mm, respectively; SP is a high-range water-reducing agent; W/C denotes the water-to-cement ratio.

**Table 3:** Parameters of the SE-MCNN.

Scale 1	Scale 2	Scale 3
Convolution (1 × @16)	Convolution (1 × 7@16)	Convolution (1 × 7@16)
Pooling (3)	Pooling (3)	Pooling (3)
Convolution (1 × 5@32)	Convolution (1 × 5@32)	Convolution (1 × 5@32)
Pooling (3)	Pooling (3)	Pooling (3)
Convolution (1 × 3@64)	Convolution (1 × 3@64)	Convolution (1 × 3@64)
Pooling (3)	Pooling (3)	Pooling (3)
Concatenate (1 × 1@64)		
Fully connecting (64, 3)		

**Table 4:** Statistical table of classification results.

Signal Label	Number of Signal Groups	Time of Duration
0	1564	39.100 s
1	582	14.55 s
2	381	9.525 s

## Data Availability

Data Sharing is not applicable.
